# Rapid SARS-CoV-2 Antigen Detection Self-Tests to Increase COVID-19 Case Detection in Peru: Qualitative Study

**DOI:** 10.2196/43183

**Published:** 2023-03-17

**Authors:** Paola A Torres-Slimming, Cesar Carcamo, Guillermo Z Martínez-Pérez, Patricia Mallma, Cristina Pflucker, Sonjelle Shilton

**Affiliations:** 1 Universidad Peruana Cayetano Heredia Lima Peru; 2 FIND, the global alliance for diagnostics Geneva Switzerland

**Keywords:** Peru, COVID-19, self-testing, diagnostics, qualitative research, testing, virus, detection, health, decision-making, public, willingness, health system

## Abstract

**Background:**

The COVID-19 pandemic heavily impacted many low- and middle-income countries (LMICs), such as Peru, overwhelming their health systems. Rapid antigen detection self-tests for SARS-CoV-2, the virus that causes COVID-19, have been proposed as a portable, safe, affordable, and easy-to-perform approach to improve early detection and surveillance of SARS-CoV-2 in resource-constrained populations where there are gaps in access to health care.

**Objective:**

This study aims to explore decision makers’ values and attitudes around SARS-CoV-2 self-testing.

**Methods:**

In 2021, we conducted a qualitative study in 2 areas of Peru (urban Lima and rural Valle del Mantaro). Purposive sampling was used to identify representatives of civil society groups (RSCs), health care workers (HCWs), and potential implementers (PIs) to act as informants whose voices would provide a proxy for the public’s attitudes around self-testing.

**Results:**

In total, 30 informants participated in individual, semistructured interviews (SSIs) and 29 informants participated in 5 focus group discussions (FGDs). Self-tests were considered to represent an approach to increase access to testing that both the rural and urban public in Peru would accept. Results showed that the public would prefer saliva-based self-tests and would prefer to access them in their community pharmacies. In addition, information about how to perform a self-test should be clear for each population subgroup in Peru. The tests should be of high quality and low cost. Health-informed communication strategies must also accompany any introduction of self-testing.

**Conclusions:**

In Peru, decision makers consider that the public would be willing to accept SARS-CoV-2 self-tests if they are accurate, safe to use, easily available, and affordable. Adequate information about the self-tests’ features and instructions, as well as about postuse access to counseling and care, must be made available through the Ministry of Health in Peru.

## Introduction

On March 11, 2020, the World Health Organization (WHO) declared COVID-19 a global pandemic [1]. By mid-February 2022, a cumulative total of 420 million cases had been reported [2]. Worldwide, rapid efforts were made to limit the spread of COVID-19. After more than 2 years of the COVID-19 pandemic, public health–related measures worldwide have included travel restrictions, social distancing, personal protective measures, and strict quarantines. Public health systems have faced various obstacles, including the surveillance and reporting of new variants of concern (VoCs), such as the Omicron VoC [3].

Peru reported 974,887 cases of COVID-19 and 80,854 deaths during its first epidemic wave and 1,231,617 cases and 97,951 deaths during its second epidemic wave [4]. The restrictions implemented in Peru, as in many low- and middle-income countries (LMICs), had devastating impacts on the country’s economy [5]. The restrictions proved unacceptable, especially for those earning a living in nonregulated informal economies [6].

At the beginning of the pandemic, serological tests and real-time reverse transcription polymerase chain reaction (RT-PCR) were available only from the Peruvian Ministry of Health (Ministerio de Salud [MINSA]). At the time of the second epidemic wave, MINSA authorized other private institutions to provide COVID-19 testing for contact-tracing purposes, as well as to accelerate access to treatment for infected individuals [7]. RT-PCR is the gold standard for the detection of SARS-CoV-2, the virus that causes COVID-19 [8]. However, although RT-PCR is highly accurate, it is not a cost-effective solution for mass screening programs.

Rapid antigen detection tests (RATs) for SARS-CoV-2, by means of lateral flow tests, are point-of-care assays; in September 2020, WHO authorized them for emergency use [9]. RATs are qualitative antigen detection assays that can also be used for self-testing. According to the European Centre for Disease Prevention and Control (ECDC), SARS-CoV-2 self-testing can complement provider-initiated testing and facilitate earlier detection of COVID-19 cases [10].

The Government of India [11], the Brazilian Regulatory Authority [12], and the African Union [13], among other organizations, recommend SARS-CoV-2 self-testing (hereafter, self-testing) as a complementary approach to improve a population’s access to SARS-CoV-2 testing. Prior to any scale-up of self-testing alongside conventional screening approaches, the views, preferences, and attitudes toward self-testing of the populations concerned must be assessed. Such knowledge is crucial to ensure that health authorities can deploy self-testing in a safe way. With the aim of informing WHO policy guidance and Peru’s public health authorities, a qualitative study was conducted to investigate the Peruvian public’s values and preferences around self-testing.

## Methods

### Study Design

This qualitative investigation was led by the Universidad Peruana Cayetano Heredia, Peru, in collaboration with FIND, the global alliance for diagnostics. The research involved the use of semistructured interviews (SSIs) and focus group discussions (FGDs) as data generation techniques. This research followed a thematic comparative analysis approach.

Two study sites in Peru were selected, the urban city of Lima and rural areas of the Valle del Mantaro (Junín region). Although Lima was selected with the aim of gaining insights from decision makers based in an urban area, the Valle del Mantaro was chosen as a rural area due to its sociodemographic, professional, and economic differences to Lima and because it was perceived to be an appropriate setting to ensure a diversity of voices in the study sample.

This study was conducted using the frame of a multisite, mixed methods evaluation of the general public’s values and attitudes toward self-testing that was conducted in 8 LMICs during 2021 [14].

### Study Population

Three subpopulations were targeted as study groups. Health care workers (HCWs; eg, nurses, physicians) were targeted because of their capacity to recommend self-testing to their patients. Representatives of civil society groups (RCSs; eg, traditional community leaders, representatives of trade unions or professional councils, spokespersons of civil society organizations) were targeted because of their capacity to influence community decision-making in relation to the utility of self-testing. Potential implementers (PIs) of self-testing delivery programs (eg, leaders of private industries or large corporations, directors of departments of public health) were targeted because of their capacity to decide how to pool financial and human resources to procure and distribute self-tests at scale in the workplaces they manage or in the geographies where they have jurisdiction.

Common inclusion criteria for all populations were being aged 18 years or older, fluency in Spanish, and willingness to provide informed consent.

### Sampling and Recruitment

The recruitment of study informants and data collection were conducted between August and December 2021. Google, LinkedIn, and institutional websites were used to identify as many people as possible who met the inclusion criteria and the definition of the study populations. Google Maps and social media (Facebook, Instagram) were especially useful for identifying nongovernmental, profit, and nonprofit organizations operating in the Valle del Mantaro.

The study team proposed a sex-disaggregated list of up to 50 profiles per study group, which had been purposively selected with the aim of covering a variety of social, professional, and institutional profiles. These lists were randomly rearranged using RANDOM.org, a random list generator. Starting with the first name in each randomized list, potential informants were then contacted by phone, informed about the study, and, if they were interested in participating, asked to participate in either an SSI or an FGD.

### Data Collection

In pursuing data saturation, a minimum of 30 SSIs (10, 33%, with each of the 3 study groups) and 6 FGDs (2, 33%, with each of the 3 study groups) were proposed. As data collection and analysis were to occur in a contemporaneous manner, this target size was to be increased in the event that data saturation was considered unachieved by the data analysis team.

Data collection was conducted by a medical epidemiologist (female, PhD) and a sociologist (male, MA). Both were Peruvian and had experience in qualitative research. Due to the COVID-19 pandemic, some informants expressed a preference to participate in online SSIs conducted via Zoom software. However, when in-person interviews were possible, data collection was conducted at a designated place convenient for the informant and the interviewer. The meeting places were all outdoors, with the aim of increasing the physical safety of the informants and interviewers.

A 45-item structured guide was used for both SSIs and FGDs. The items were organized into 6 themes: (1) knowledge of provider-initiated COVID-19 testing, (2) values in relation to self-testing, (3) preferences for the delivery of self-testing, (4) safe and effective use of self-testing, (5) likely actions upon receiving results of self-testing, and (6) prospects for future distribution of self-testing. The items were to be explored sequentially by the interviewers. Within each item, there were some open-ended questions that could be explored further, depending on the nature of the informants’ responses.

### Data Analysis

The SSIs and FGDs were audio-recorded. The recordings were transcribed verbatim. Verified transcripts were coded in Quirkos. At first, all transcripts were coded deductively using a predefined coding scheme. Whenever an emerging theme was identified, a new code was created inductively. In parallel to coding, the analyst prepared reflexive memos to avoid informant bias. Field notes were included with the memo process to contextualize the analysis.

A thematic analysis was applied, following a 4-stage approach: (1) transcript by transcript, (2) a theme-by-theme sex-sensitive comparison of coded narratives across all transcripts, (3) a theme-by-theme rural- versus urban-sensitive comparison of coded narratives across all transcripts, and (4) a comparison of key findings across the 3 study populations. For reporting, Consolidated Criteria for Reporting Qualitative Research (COREQ) guidelines were considered [15].

### Ethical Considerations

This study received ethical approval from the Institutional Review Board of the Universidad Peruana Cayetano Heredia, Peru (approval number: 205954). All informants signed an informed consent form and received a copy. Informants who came for the in-person FGDs and SSIs were compensated for their transportation.

## Results

### Informants’ Characteristics

In total, 30 informants participated in 30 SSIs (average 49 minutes) and 29 informants participated in 5 FGDs (average 82 minutes); see Table 1. Informants from urban areas were all from Lima (n=28, 47%), and informants from rural areas (n=31, 53%) were from the municipalities of Cullpa Alta, Palian, Cochas, and Uñas. In addition, 27 (46%) informants were male. Furthermore, 2 (3%) PIs and 1 (2%) RCS had completed postgraduate education, 1 (2%) RCS had no education, and 2 (3%) RCSs had primary education; both were from rural settings. A total of 29 (49%) informants reported having higher education, either completed or ongoing. Of the 20 HCWs, 4 (20%) were physicians who had completed their medical fellowships.

**Table 1:** Sociodemographic characteristics of the study informants (N=59).

Variable		HCWs^a^ (n=20, 34%)	PIs^b^ (n=18, 31%)	RCSs^c^ (n=21, 35%)
Age (years), median (IQR)		35.0 (28.5-43.5)	42.5 (34.0-61.0)	46.0 (35.0-56.0)
**Location, n (%)**				
	Rural	10 (50)	10 (56)	11 (52)
	Urban	10 (50)	8 (44)	10 (48)
**Sex, n (%)**				
	Female	11 (55)	11 (61)	10 (48)
	Male	9 (45)	7 (39)	11 (52)
**Education, n (%)**				
	No education	0	0	1 (5)
	Primary education	0	0	2 (10)
	Secondary education	4 (20)	5 (28)	3 (14)
	Higher education completed	4 (20)	5 (28)	3 (14)
	Higher education ongoing/incomplete	8 (40)	3 (17)	6 (29)
	Postgraduate education incomplete	4 (20)	3 (17)	5 (24)
	Postgraduate education complete	0	2 (11)	1 (5)

^a^HCW: health care worker.

^b^PI: potential implementer.

^c^RCS: representative of civil society group.

### Uptake of COVID-19 Testing

The informants stated that any individual with COVID-19–related symptoms should take a test from any of the 3 available tests (serological tests, RATs, or RT-PCR) offered by the Peruvian health system or private outlets. Two of the urban PIs working in the public health sector considered any of these 3 types as acceptable to confirm a COVID-19 diagnosis, although most RCSs were unable to explain the differences between these 3 available COVID-19 tests. However, the majority of the HCWs explained that only RT-PCR or an RAT could be used to confirm a diagnosis of a current SARS-CoV-2 infection.

Most HCWs specified that any suspected case of COVID-19 should be isolated, even in the absence of testing, and monitored for symptoms until an antigen detection test could be performed. It was explained that this process was facilitated by the opening of well-equipped private COVID-19 testing centers certified by Peru’s National Institute of Health (Instituto Nacional de Salud [INS]). However, as reported by some PIs, testing sites in rural areas were scarce, even though there was a need for testing, as some rural areas are frequently visited by travelers, truck drivers, and merchants.

They [COVID-19 tests] are requested for travel, for work, and on some occasions where you have been in contact with the general public (…) or in a risky situation.

Urban male RCS, 44 years old

HCWs and PIs with experience in the health sector noted that at the beginning of the pandemic, there was less access to COVID-19 testing. Initially, serological tests were the only tests available, and their use was restricted to MINSA facilities. Subsequently, RATs and RT-PCR were introduced, and their production was regulated by the INS. In those early days, reportedly, any public or private laboratory could collect samples from suspected cases, but the samples had to be sent to the INS. As the pandemic evolved, more laboratories were authorized to conduct COVID-19 tests themselves.

A reported barrier to COVID-19 testing was the discomfort of the procedure, which involved the collection of nasopharyngeal swabs and was considered invasive and painful. The cost of tests, the lack of human resources, and the scarcity of laboratory supplies were other described deterrents to both testing uptake for individuals and for MINSA to implement mass testing.

As government testing policies evolved, the demand for tests from the private sector increased for traveling, work, and mass screening. Most informants agreed that in addition to costs, the “fear of knowing the result” was a factor that limited many individuals’ willingness to demand testing. A fear of awareness of being positive for SARS-CoV-2 was associated with the fear of death and disease but also with the fear of losing a job, of incurring health care expenditure, and of being rejected by friends or family. This was found to be associated with a sense of guilt for having acquired SARS-CoV-2. An urban female RCS attributed this sentiment of guilt to the Peruvian government’s commentary on the disease:

At that time, there was a sense of shame incentivized by the government and the media, where if you got sick it was because you were irresponsible, because you did not comply with the rules, you were the culprit of your own illness.

Urban female RCS, 57 years old

All the HCWs explained that they had worked on COVID-19, either in population-based screening campaigns or in laboratory-based testing centers. Most HCWs mentioned that at the beginning of the pandemic, there was less use of diagnostics to rule out COVID-19, due to a lack of tests and their high costs. An urban HCW stated that the inefficient distribution of RATs at the beginning of the pandemic symbolized how Peru’s public health care system had been progressively “mutilated”:

In our country we have mutilated primary health care (…) that is why second and third level [hospital] services were saturated.

Urban male HCW, 54 years old

Most informants considered it necessary that provider-initiated COVID-19 tests be performed in symptomatic cases, for close contacts, and to facilitate a return to socioprofessional activities. Some rural informants expressed that in rural areas, there was less ability to carry out mass screening tests to confirm positive cases. Therefore, in rural areas, it was suggested that it would be useful if self-tests were available in pharmacies, rather than in health centers, as pharmacies are widely distributed and could cover rural populations’ needs for testing.

Many informants commented that at the beginning of the pandemic, the individuals who were “scared of dying alone in a hospital” were the most reluctant to go to the hospital to request testing. When more tests became available, several HCWs mentioned that their patients were hesitant to get tested for fear of missing a workday. Furthermore, if they tested positive, they perceived that a sense of stigma would accompany them. Such was the case reported by a female rural physician. While conducting her work in a community near the city of Huancayo in Valle del Mantaro, she observed the effect in this community, with the presence of COVID-19 health emergency groups or *brigadas* (in Peruvian Spanish, *brigada* means brigade). Families where these brigades entered their household for case screening experienced discrimination within the community.

Some informants considered that barriers to testing might have now diminished as a result of the increased availability of accurate information about COVID-19. Even so, it should be noted that as the RCSs and HCWs mentioned, in rural areas particularly, sentiments of fear of discrimination and disbelief of official information about COVID-10 led some rural inhabitants to use traditional medicine. Rural HCWs indicated that these local practices are sometimes endorsed by health care professionals. For example, during the pandemic, there was inappropriate use of chlorine and ivermectin, as well as herbs thought to have healing effects, such as eucalyptus, *matico* (*Piper aduncum*), and *puchishpay*.

Puchishpay is human urine, led to stand and rot for a month or even up to 6 months (…) Even my own brother, who was desperate: he boiled the urine with some herbs and eucalyptus. He took a bath and drank a complete bowl (…) The next day he was doing well (…) Even death turns away because of puchishpay.

Rural mixed-sex FGD with PIs

### Self-Testing Preferences and Barriers

Only a few informants knew that self-tests were in use abroad. When informants were presented with images of self-tests and asked to explain what they were, just a few urban informants recognized the devices, having seen them before in pharmacies and other stores on trips to the United States. However, no informant had ever used a self-test themselves or knew of anyone who had. Despite this, they were all interested in introducing self-testing to the Peruvian market. Their rationale was that it would accelerate diagnosis and reduce costs for users.

Two of the rural HCWs pointed out the advantages of self-testing for monitoring epidemic waves of COVID-19, preventing the collapse of health systems due to rapid increases in case numbers. In general, self-testing was perceived to be an innovation that would improve access to testing for poor and vulnerable populations. Furthermore, it would allow users greater flexibility by self-testing at home rather than having to travel long distances to health establishments.

An urban male RCS who mentioned having experiences in relation to HIV self-testing considered self-testing for SARS-CoV-2 to be a great initiative, as many people were reluctant to attend a health facility for testing, often due to fear, discrimination, or ignorance about the availability of tests or their remoteness from a facility.

Yes, indeed it is a very positive measure. We have already experience massive self-testing for HIV. What we hope for is that populations can access earlier to health services or, for instance, know what to do about a positive test, but if you do not know what measures to take, overcome, or what type of COVID-19 you have, you rather feel more scared, and that [feeling] we should avoid.

Urban male RCS, 44 years old

Regarding access to tests from outlets other than health care facilities, markets, pharmacies, and drugstores were mentioned as being adequate. The specific reasons given were that the health system would benefit from reduced costs and effort spent in training health workers, hence reducing the burden on the health system.

Most informants were clear that any self-tests entering the Peruvian market must be regulated and quality controlled so as not to bring in *bamba* products (products of poor quality). A few informants mentioned that self-testing should have reliable accuracy, ideally not less than 90% for both sensitivity and specificity. A 40-year-old urban female RCS stated that self-testing devices should at least provide greater benefits than serological and antigen tests. In her opinion, a lower sensitivity and specificity than PCR could be acceptable if the speed of obtaining a result partnered with a low cost compensated for their decreased accuracy. Clear information about a self-test device’s accuracy was considered crucial by many rural RCSs, who commented that in rural areas, some people might have a fear regarding the possibility of false results. This was, in their opinion, due to exposure to contradictory messages from health personnel working in rural areas who expressed concerns about the reliability of rapid serological or antigen detection tests.

The informants suggested that self-testing should be made available for the entire population. Some informants specified that the target population for self-testing should be secondary- and university-level students, as young people are usually up to date with technology. Rural informants mentioned that individuals with the most economic resources would be those most interested in self-testing. As an urban male RCS explained:

It is for the more educated, those who have more knowledge and that could be [available for] him and his family. So, I think the advantage would be that you can have a quick diagnosis when you have suspicious symptoms or when you have been in contact with patients who are already diagnosed, right?

Urban male RCS, 40 years old

Most rural RCSs, who considered themselves to be representatives of their communities, expressed their willingness to share information about self-testing:

We here in our neighborhood, and as a leader that I am, I have all the desire [intention] and responsibility to safeguard the integrity, the health of all our neighbors, because if we take care of ourselves and others, we take care of our entire district.

Rural female RCS, 44 years old

All PIs were interested in the applicability of self-testing for the general population, including at the family level, for adolescents, and for older adults. An urban male PI stated that it would be useful to distribute self-tests among the most vulnerable populations, such as health personnel and school children. He emphasized that in remote areas, health workers could use self-tests as a complement to RATs. Another 45-year-old rural male PI thought that women might be keen to use self-tests as they are the ones who take care of health at home.

All informants suggested that self-testing should not be based on nasopharyngeal swabs in the way that RATs and RT-PCR are. Many considered that as the collection of nasopharyngeal swabs is a difficult procedure even for trained staff, this might be challenge for laypersons. The main disadvantage noted was the inadequate placement of the swab, in addition to the discomfort and pain experienced by the recipient.

A 57-year-old urban female PI commented on the necessity of introducing self-testing in LMICs. However, she recognized there are multiple regulatory barriers and mentioned the power held by laboratories and medical doctors, in the sense that it is difficult for this group to accept innovative approaches if this implies a loss of their “status or paternalistic position and in laboratories’ economic gains.”

Some HCWs explained that in Peru, many physicians wish to deliver health at the individual level, not considering public health interventions or pandemic scenarios. In their opinion, many physicians are unwilling to empower the population in the use of self-testing devices or lack knowledge about how to interpret self-test results.

In my experience with HIV rapid tests, not all clinicians understand the concepts of sensitivity, specificity and predictive values.

Urban female PI, 57 years old

This concern was further discussed by HCWs during the FGDs. In these encounters, some male physicians considered that only HCWs may benefit from self-testing by testing themselves regularly if they are exposed in their workplace. They also considered that the correct use of self-tests could only be achieved if supervised by physicians, otherwise members of the public may rely on false-negative results. However, a male HCW participating in an FGD had the opposite opinion: based on his experience, self-testing could improve surveillance and avoid further hospitalizations.

### Safe and Effective Strategies for Using Self-Tests

Regarding the cost, informants considered an acceptable price to be in the range of Peruvian soles (PEN) 15.00 (approximately US $4.00) to PEN 40.00 (approximately US $10.50). A female urban PI who worked with vulnerable populations suggested that self-tests should cost less than PEN 10 (US $2.65) or even be free of charge. One male urban PI and a female urban HCW were the only informants who supported free self-tests for the public. In tackling potential concerns about the affordability of self-testing devices for the general public, a female urban RCS explained how facilitating free access to self-testing for poorer populations would be necessary:

If I work today, today I have an income. If I don’t work, there is no income at all. Many people rather pay no attention to isolating themselves because they need to earn an income. So, rather look for strategies which can support these people [in extreme poverty].

Urban female RCS, 57 years old

In relation to the place of sale, pharmacy chains were proposed. PI and RCS informants considered vending machines in supermarkets or kiosks to be possible alternatives. However, the issue of humidity and temperature control for the correct storage of the tests could be a limitation.

Informants considered that self-tests must be certified and have QR code links to access information about the product. Users’ doubts about the self-tests could be resolved via a helpline, but some informants stated that a health professional, either a nursing technician assistant or a nurse, should staff the helpline. If someone required assistance with a self-test, many informants stated that pharmacies could have personnel trained to supervise self-test users. Instructions for use that are easy to understand and interpret would reduce the chance that users would make errors or need assistance.

The informants expressed that primary-level health centers and public and private hospitals would be essential for the delivery and supervision of self-tests. Likewise, pharmacies and drugstores would be important in remote, underserved, and geographically excluded places. A rural HCW suggested using an approach similar to the population-screening campaigns conducted by the Peruvian Red Cross.

A situation that warrants considerable concern is the distribution and storage of self-tests. If self-tests became available on the black market, this could lead to the sale of products past their expiration date or that had been inadequately stored.

The only inconvenient might be that it can be sold by a profit, and then resold, as it happens with game tickets (…) Once it is bought at low prices, it is taken and stored in warehouses (…) It is easy to transport and create black parallel markets.

Rural male PI, 50 years old

It was suggested by most rural informants that at the rural level, cooperation with local municipalities, local leaders, or trained health promoters would be essential to socialize information about how to access and correctly use self-tests. As an urban RCS mentioned, any distribution of self-tests by private companies or nongovernmental institutions must be supported by MINSA.

Information is one, second is prices, and the last is coordination between health care facilities and pharmacies.

Urban male RCS, 50 years old

Once self-tests are available, information campaigns should be conducted regarding their use and actions to take upon receiving a positive result. Thus, if a person tests positive, the actions to be followed must be directed at (1) their own care and monitoring of their symptoms and (2) reporting their result and tracing of possible contacts. To achieve these 2 aspects, the PIs and HCWs suggested the tests must have a QR code system to allow results to be entered into a database for epidemiological surveillance at the national level.

Most informants suggested that it is the responsibility of the self-test user to notify their contacts. Some informants believed that upon learning their result, individuals would only share this with their relatives or with those with whom they live. An urban female PI who worked with remote rural communities commented that some people have had COVID-19 and “take it like a cold.” In her experience, it was highly unlikely that people in those communities would report a positive result.

As for the actions that people might take following a positive self-test result, informants thought that this would depend on the severity of the individual’s symptoms and with whom the person lived. For example, if users live alone and can work remotely, they would voluntarily isolate. In general, the informants believed that few people would be likely to observe strict isolation. They argued that this was because much of the population relies on day-to-day work. This relates to “a lot of informal jobs,” which do not cover sick days.

In addition, due to financial constraints, many people often delay going to a health center to the point that their oxygen saturation levels are too low. An interesting point mentioned by a female rural PI related to strict prohibitions. This was especially true in the city of Huancayo and surrounding areas, where families were not able to visit sick relatives or watch over their dead. These drastic measures, especially at the beginning of the pandemic, led to people choosing to isolate themselves, not discussing their symptoms, staying at home, and not attending health services for confirmatory testing.

### Prospects for Future Distribution of Self-Testing

In general, informants were critical of the success of COVID-19 control measures and campaigns. Hence, many informants expressed the need to improve any future self-testing delivery programs by providing support at the community level through local leaders, municipality representatives, and the national police. However, some informants, especially the HCWs, also recognized that it would be useful to conduct epidemiological surveillance with the participation of communities to help prevent future outbreaks.

Regarding the regulation and standards of any self-tests, most PIs mentioned that the tests should be certified by WHO and MINSA. The easiest way for people to access self-tests would be at *boticas* (in Peruvian Spanish, *botica* means chemist or small pharmacy or drugstore), as these outlets can work in coordination with primary health care centers. The more self-testing becomes available on a mass scale and accessible at the population level, the more the population will perceive it to be an acceptable alternative to enhance the surveillance of COVID-19. Regarding instructions for use, these must be easy to understand. Furthermore, to guarantee mass accessibility, self-tests must be affordable or free of charge.

As an alternative to cover costs, the insurer Institution Provider of Health Services (Institución Prestadora de Servicios de Salud [IPRES]) could pay for self-testing or the state could subsidize the cost. A low cost may be a facilitator for the mass use of self-tests, but it must be accompanied by adequate information campaigns. Informative advertisements, webinars, talks at the health center level, or campaigns should be provided. Another option would be via television programs or radio spots. The latter might be best for rural settings. Another alternative would be to use Peruvian celebrities, such as soccer players, to promote self-testing.

It was suggested by a few informants that all scientific information around self-testing should be supported and confirmed by MINSA so that the public can determine its validity. Hence, a self-test must be accurate to gain the public’s trust. In addition to publicizing self-tests, the usefulness of self-testing and what to do in the event of a reactive result should be highlighted. A joint effort between MINSA, the municipalities, and the civil society was suggested for epidemiological surveillance, caring for positive cases and their contacts. An urban female PI mentioned the need to educate health providers in “self-care-initiated interventions.” For instance, health professionals should understand that they will not lose power by empowering the population to supervise their own health. As recommended by this PI, it should be a priority to understand the barriers among various health care providers to developing effective health communication responses.

Look, I have taken my self-test: I am with these symptoms, it came out positive or negative. What to do next?

Male urban PI, 41 years old

In general, the proposed route for acquiring a self-test was as follows: An individual could go to the nearest place (preferably a *botica*) and buy the device. They could either request advice on how to use it at the same place or read the instructions on their own at home. In the case of questions, there could be telephone hotlines for end users to resolve their doubts. There was just 1 deviant case, an urban 48-year-old PI, who was completely against self-testing in LMICs, as they considered that any public health measures involving self-testing should be supervised exclusively by well-trained health personnel from MINSA.

## Discussion

### Principal Findings

This study explored decision makers’ values and preferences around SARS-CoV-2 self-testing in Peru. Most of the study informants did not know about self-testing; however, they appreciated its advantages and the benefits that it could bring, both to the general public and to the public health system in Peru. The majority of informants expressed a willingness to recommend its use. Regarding preferences, it was suggested that self-testing be delivered in local pharmacies in coordination with public health services. In the informants’ opinion, if self-test devices had a price of between US $5.00 and US $10.00, the public might find this cost acceptable. These findings are aligned with the results of other qualitative studies into self-testing conducted in low- and middle-income settings, such as Indonesia [16] and South Africa [17]. Additionally, in 2 population-based surveys conducted in parallel with our qualitative study, in Kenya [18] and Indonesia [19], 81.4% and 60.8%, respectively, of the general public expressed a willingness to use self-testing, and 63.0% and 62.1%, respectively, expressed a willingness to pay for a self-test device.

Narratives from our informants suggest that inadequate access to and understanding of information about COVID-19 diagnostics during the evolution of the pandemic could have shaped people’s views, behaviors, and perceptions around COVID-19 testing. Misunderstandings and knowledge gaps in relation to COVID-19 testing might affect the future of self-testing as a complement to provider-initiated testing in Peru [20,21]. A study in the United States into the perceptions of individuals undertaking self-testing suggested there are benefits to providing clear information around the accuracy of self-test devices [22]. Although the social fabric and cultural environment in Peru are different to those in the United States, it would be reasonable to assume that clear information around the accuracy of self-test devices might also help the Peruvian public perceive that self-testing is a safe and reliable alternative to seeking testing in congested facilities. Nevertheless, the conflicting messages that placed a strain on public health interventions during the pandemic must be avoided in any future delivery of self-testing in the country. Those responsible for the delivery of self-testing in Peru need to consider, when developing information about self-testing materials targeting low-literacy groups, that the infodemic and digital health illiteracy were almost as great a problem as COVID-19 itself [23].

Communication strategies in relation to self-testing should be clear for various populations, respecting their cultural use of traditional medicine. This use has been reported among indigenous Amazonian populations, with plants widely used for protection against COVID-19, while a sense of mistrust and discomfort grew during the pandemic due to the lack of health care surveillance among these communities [24]. It has also been described how indigenous communities in the United States have combined cultural traditions with Western medicine responses to COVID-19 [25]. The results of the studies among indigenous populations concur with suggestions made by our study informants, that is, to improve health literacy, it is necessary to integrate the cultural notions of what disease prevention interventions will be considered by the public as congruent with their values and needs [26].

There is a paucity of qualitative literature relating to self-testing in low-income countries, and at the time of preparing this manuscript, a comparison of our findings with other reports in the scientific literature was not feasible. However, it should be noted that data collection for this study concluded in December 2021 and that the government of Peru approved self-testing in early 2022 [27]. Some of our informants’ insights anticipated the reactions of the public and health experts reported in both social and traditional media. Hence, it is recommended that our informants’ voices be considered when planning and programming the delivery of self-testing in Peru. As our informants lacked actual experience as either users or distributors of self-testing, further studies into the actual experiences of the public with self-testing are necessary.

### Limitations

Some limitations of our study should be noted. Social unrest following the Omicron wave in Peru might have had an effect on the informants’ narratives around the added value of self-testing. Due to concerns over the impact of the Omicron VoC, many informants were reluctant to be interviewed in person. During online data collection, it is more difficult to pay attention to a participant’s body language and other nuances that are useful for qualitative data analysis. Another limitation of the study was that despite the use of a purposive sampling approach, some potential informants in the PI and HCW groups refused to participate in FGDs in the company of other individuals they did not already know. In both rural and urban study sites, potential informants who had no online institutional or social media profiles might have been missed at the sampling stage. Snowball sampling was introduced in some instances to enable the finalization of recruitment. The introduction of this technique might have resulted in the introduction of social desirability bias in some interviews.

### Conclusion

According to informants in this qualitative inquiry conducted in Peru at the end of 2021, SARS-CoV-2 self-testing could be an acceptable diagnostic approach to complement the health system’s efforts to increase COVID-19 case detection among the public. Although the decision makers partaking in our inquiry did not have actual experience as users of self-testing, when proposed as a complement for surveillance, the majority were willing to support its use. Decision makers engaged in this qualitative study considered that the public would be willing to accept SARS-CoV-2 self-tests if they were accurate, safe to use, easily available, and affordable. In their opinions, adequate information about the self-tests’ features and instructions, as well as about postuse access to counseling and care, must be made available through the Ministry of Health in Peru.

## Abbreviations

FGD: focus group discussion
HCW: health care worker
INS: Instituto Nacional de Salud (National Institute of Health)
LMIC: low- and middle-income country
MINSA: Ministerio de Salud (Ministry of Health)
PEN: Peruvian soles
PI: potential implementer
RAT: rapid antigen detection test
RSC: representative of civil society group
RT-PCR: reverse transcription polymerase chain reaction
SSI: semistructured interview
VoC: variant of concern
WHO: World Health Organization

## Data Availability

The data sets for this study cannot be found in any repository. This is qualitative research; hence, to protect the anonymity, privacy, and confidentiality of the study informants, the data sets cannot be made public.
